# Consumer Behavior in Shopping Streets: The Importance of the Salesperson's Professional Personal Attention

**DOI:** 10.3389/fpsyg.2016.00125

**Published:** 2016-02-10

**Authors:** Natalia Medrano, Cristina Olarte-Pascual, Jorge Pelegrín-Borondo, Yolanda Sierra-Murillo

**Affiliations:** Departamento de Economía y Empresa, Universidad de la RiojaLogroño, Spain

**Keywords:** consumer behavior, small retail, mall, shopping street, expectations, personal attention, personal relationship

## Abstract

Since the early 2010s, the emergence of a new consumer has begun. In this context, consumer behavior represents one of the greatest interests of marketing scholars and business managers due to their need to adapt their companies' strategies to the new frontier. In order to advance understanding of this new consumer, this article focuses on analyzing consumer behavior in shopping streets. Thus, the aim of this research is to know what customers value in terms of salesperson–customer interaction quality nowadays. To achieve this, the authors conducted two studies. The results of the first study show that customers cite personal attention as the primary factor motivating their preference for small retailers in shopping streets. However, this motivation is not as relevant one for those who prefer malls. This result provides a point on which to research service quality incorporating personal attention in a second study. Using the SERVQUAL-P scale, the authors elaborate three lenses through which the quality of service from the customer's point of view can be analyzed: normative expectations, predictive expectations, and the importance of each attribute. The most striking result is that the dimensions of expectations (normative and predictive) are the same; these results demonstrate that customers are coherent in making assessments of their expectations, evaluating service quality and satisfaction with similar criteria. However, these dimensions are different from the dimensions of importance. Our main contribution lies in the finding that personal attention, when assessed using the scale of attribute importance, is split into two dimensions: (1) courteous attention and (2) personal relationship. Courteous attention is always welcome, but personal relationships are less valued and are often even rejected. The article concludes with a discussion of the implications of these findings for marketing practices and research.

## Introduction

Cities in Europe are often characterized by an urban center with commercial streets in which numerous independent stores are located. These stores are often family-owned, small, and specialized, and the employees tend to have an in-depth knowledge of the product and a greater focus on customer service. Commercial development, in contrast, has been characterized by the emergence of large malls located on the outskirts of cities. These malls have led to the displacement of consumers toward these urban peripheries, thus hurting the more traditional urban retail trade (Sadahiro, 2000; O'Callaghan and O'Riordan, 2003; Hernández and Jones, 2005). Small retailers located in shopping streets are losing customers every day; these stores eventually close, and over time cities slowly begin to lose their cultural and economic vibrancy. This has led European public authorities to take action to improve the management of their cities' commercial centers and the shops therein (Medway et al., 2000; Paddison, 2003). It could be said that traditional urban small trade of shopping streets has become an endangered species.

At the same time, since the early 2010s the emergence of a new consumer has begun: Consumer 3.0. The influence of sociocultural shifts on this consumer's purchase behavior is highlighted, especially factors that are technological, social, or emotional in nature (Sersland, 2015). Today's consumers want to feel more in control and they want to be seen and valued more than their money. Technology has radically changed the psychology of these new consumers and created a host of new expectations. They do not want to sift through irrelevant information, lengthy explanations, or anything not immediately important. In this context, consumer behavior, which is sometimes guided by self-related motives rather than by rational economic considerations (Cisek et al., 2014), represents one of the greatest interests of business managers due to their need to adapt their companies' strategies to the new frontier. Current research (Grewal et al., 2009) stresses that survival in today's economic climate and competitive retail environment requires more than just low prices and innovative products. Several authors (Badgett et al., 2007; Gentile et al., 2007; Grewal et al., 2009; Tynan and McKechnie, 2009; Verhoef et al., 2009; Rose et al., 2012) emphasize the importance of the shopping experience when choosing among different retailers.

**Figure 1:**
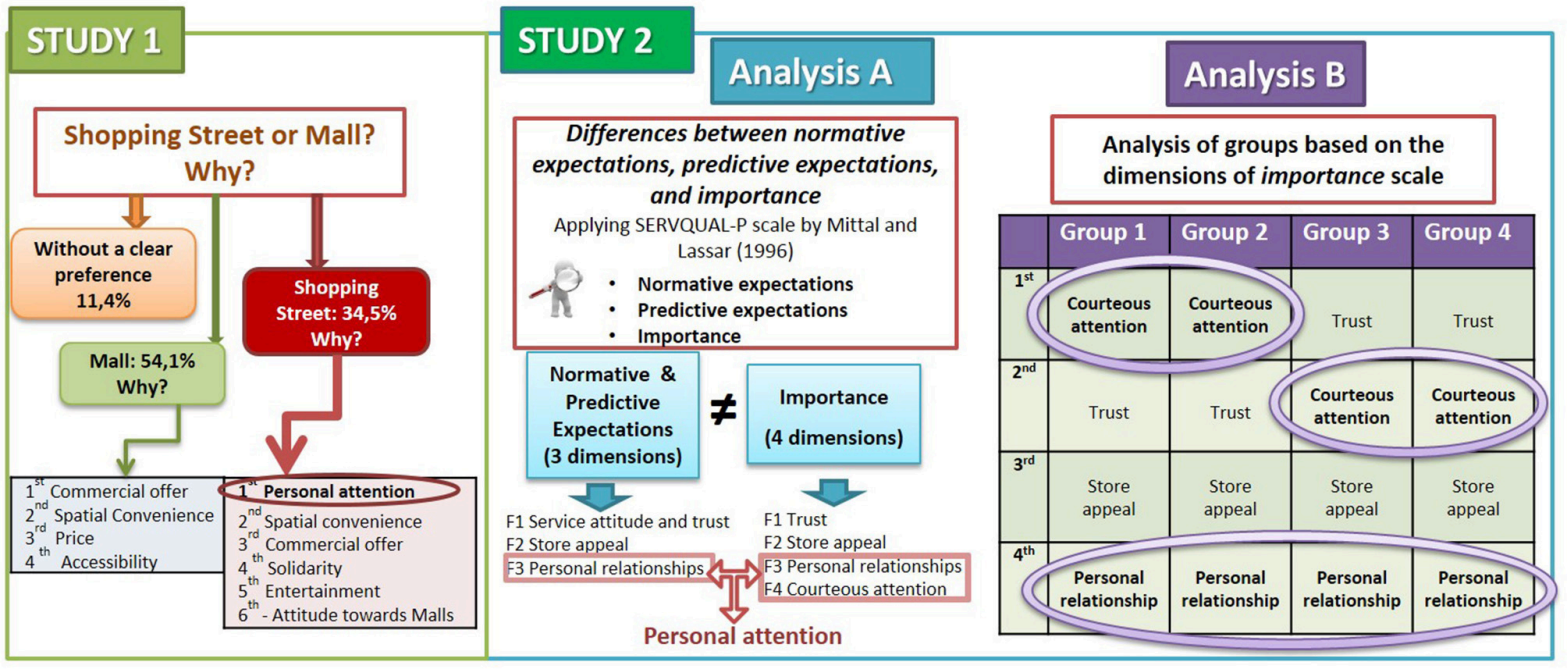
**Research diagram**.

The quality of service and the degree of personal attention are important factors in consumer behavior (Gremler and Gwinner, 2008). Thus, the aim of this research is to know what customers value in terms of salesperson–customer interaction quality nowadays. To achieve this, the authors conducted two studies (Figure 1).

Study 1 is based on 220 surveys. We find that customers perceive clear differences between malls and shopping streets. Furthermore, 54% of respondents prefer to do their shopping at malls mainly because of their wide and varied commercial offerings, and 35% prefer going to shopping streets because of their personal attention. The remaining respondents (11%) reported liking both types of retail destination and spread their purchases out between the two on the basis of price, product category, and/or convenience. It is evident from these responses that the kind of personal attention found in shopping streets is valued enough to win the loyalty of a large group of consumers.

In Study 2, we examine in greater detail how personal attention can provide a competitive advantage and explore the fundamental components of personal attention. This study is based on information obtained from a sample of 974 customers of small retailers in shopping streets. We use Mittal and Lassar's (1996) SERVQUAL-P scale, which incorporates aspects of personal attention, and apply it in three different ways. Specifically, we employ three lenses through which to view the components of personal attention: (1) “what should be” (16 attributes of service quality for customers, four of which are specifically related to personal attention) (i.e., “normative expectations”), (2) “what customers really expect” to happen at the store (i.e., “predictive expectations”), and (3) “how important each attribute is.”

In Study 2 analysis A, we demonstrate that customers differentiate between normative and predictive expectations and analyze the gap that occurs between them as well as their dimensions. The most striking result is that the dimensions of expectations (normative and predictive) are the same; however, these dimensions are different from the dimensions of importance. Our main contribution lies in the finding that personal attention, when assessed using the scale of attribute importance, is split into two dimensions: (1) courteous attention and (2) personal relationship. Courteous attention is always welcome, but personal relationships are less valued and are often even rejected (Study 2 analysis B).

The current research focuses on the Spanish city of Logroño. In 1997, it was acknowledged as the first commercial city in Spain. Currently, its commercial area includes two malls (characterized by having one or more large retailers that are a driving force of the city's economy and a city center with several shopping streets that are connected but have no large commercial spaces).

This study was approved by the University of La Rioja Research Ethics Board and according to ICC/ESOMAR *International Code on Social Research*. Each participant provided informed consent.

## Study 1: shopping street or mall? why?

### Literature review

#### The customer experience

Customer orientation is considered a competitive strategy for smaller service enterprises (Polo Peña et al., 2011). Creating a superior customer experience seems to be a central objective in today's retailing environment. A recent IBM report identifies customer experience as a key factor for companies in building loyalty to brands, channels, and services (Badgett et al., 2007). Effective retail management strategies have been linked to the creation of customer experience, which in turn leads to successful performance outcomes (Gentile et al., 2007; Grewal et al., 2009; Tynan and McKechnie, 2009; Rose et al., 2012). Yet, despite practitioners' recognition of the importance of the customer experience, the academic marketing literature on this topic has been limited. Publications on customer experience are mainly found in practitioner-oriented journals or management books.

Previous research on “customer experience” (e.g., Verhoef et al., 2009) recognizes the importance of past customer experiences, store environments, service interfaces, and store brands on future experiences. Some literature on retail experience has focused on store atmospherics and the impact of scents, music, tactile input, and color on customers' affective responses to a retailer (Naylor et al., 2008). Puccinelli et al. (2009) examine store atmospherics and the social environment. Retail environmental factors, such as social features, design, and ambience, can result in enhanced pleasure and arousal (Mehrabian and Russell, 1974; Baker et al., 1992). Other research in this area examines the presence and age of other consumers in the retail or service setting (e.g., Thakor et al., 2008) and the effect of crowds, music, and lighting (Baker et al., 2002). Novak et al. (2000) investigate the impact of website design on the customer's experience. Other research topics within the customer experience domain are personal relationships and service quality expectations.

#### Personal attention

Mittal and Lassar (1996) define personalization as the social content of interaction between service employees and their customers. In this sense, “personalization” pertains to the service employee's manner of relating to the customer on a human level: cold and impersonal at one end of the spectrum and warm and personal at the other. As such, it includes aspects such as employees' politeness and courtesy, employees' attempts to get to know the customer as a person and to engage in friendly conversation, and the exhibition of personal warmth in employee behavior.

The popularity of relationship marketing stems, in part, from the assumption that building customer relationships yields positive returns in the form of customer satisfaction, loyalty, word of mouth, and purchases (Reynolds and Beatty, 1999). Moreover, interactions between retail employees and customers can have a significant impact on customers' perceptions of the organization (Tsiros and Parasuraman, 2006; Gremler and Gwinner, 2008; Lichtenstein et al., 2010; Otnes et al., 2012; Litz and Pollack, 2015). The rapport between employees and customers represents a particularly salient issue in retail businesses characterized by significant interpersonal interactions (Haas and Kenning, 2014).

Gist (1968) emphasizes that the opportunity to develop personal relationships, and therefore to give personal attention, was one of the factors leading to the emergence of the specialty store in the early nineteenth century^1^. As the specialty store has evolved, this characteristic of personal association between buyer and seller has led to the popularity of the specialty store retailer. Many of today's specialty retailers have become successful by combining this element of personalized service with a merchandise assortment geared toward a particular market segment. It has been argued that the success of Starbucks is due to its ability to create a distinctive customer experience (Michelli, 2007). Gist (1968) concludes in his study that employees from these specialty stores need to be responsive, courteous, and knowledgeable and offer prompt, individualized service as a primary distinguishing characteristic of the shopping experience. In line with this perspective, Puccinelli et al. (2009, p. 24) argue that “the interpersonal nature of the interaction between the customer and employee […] may be key to customer satisfaction in the retail environment.” Ulaga and Eggert (2006) identify service support and personal interaction as core differentiators in business relationships. An important insight from Johnson and Selnes (2004) is that firms which position themselves toward offerings with low economies of scale, such as personal services, must build closer relationships to create value.

However, the importance of personal attention is not universal and varies across different service industries and cultures and it should be taken into account in order to differentiate between shopping streets and malls. Several researchers have compared the factors that draw consumers to shopping streets and malls. Reimers and Clulow (2004) believe that malls provide greater spatial convenience than shopping streets. Teller and Reutterer (2008) establish that the commercial mix, value for money, and entertainment element influence the appeal of a shopping street and a mall. Finding one's way around more easily is mentioned as a positive aspect of malls. Store atmospherics (e.g., scent, temperature, air) are a factor in both shopping streets and malls, though they are a more intense factor in malls. The ranking of the retail mix attribute depends on consumers' expectations (Léo and Philippe, 2002). Finally, Reimers (2013) states that people generally perceive malls as more accessible when using their car to go shopping.

Due to the importance of the salesperson–customer interaction^2^ in defining the consumer's experience and ultimate satisfaction (Goodwin, 1996; Menon and Dubé, 2000; Stock and Hoyer, 2005; Schau et al., 2007; Gremler and Gwinner, 2008), and in light of our desire to explore the importance of personal attention in shopping streets vs. malls, we propose the following:

H_1_: Personal attention is the main motivating factor by customers who choose to go to shopping streets but not for those who choose to go to shopping malls.

### Data

**Figure 2:**
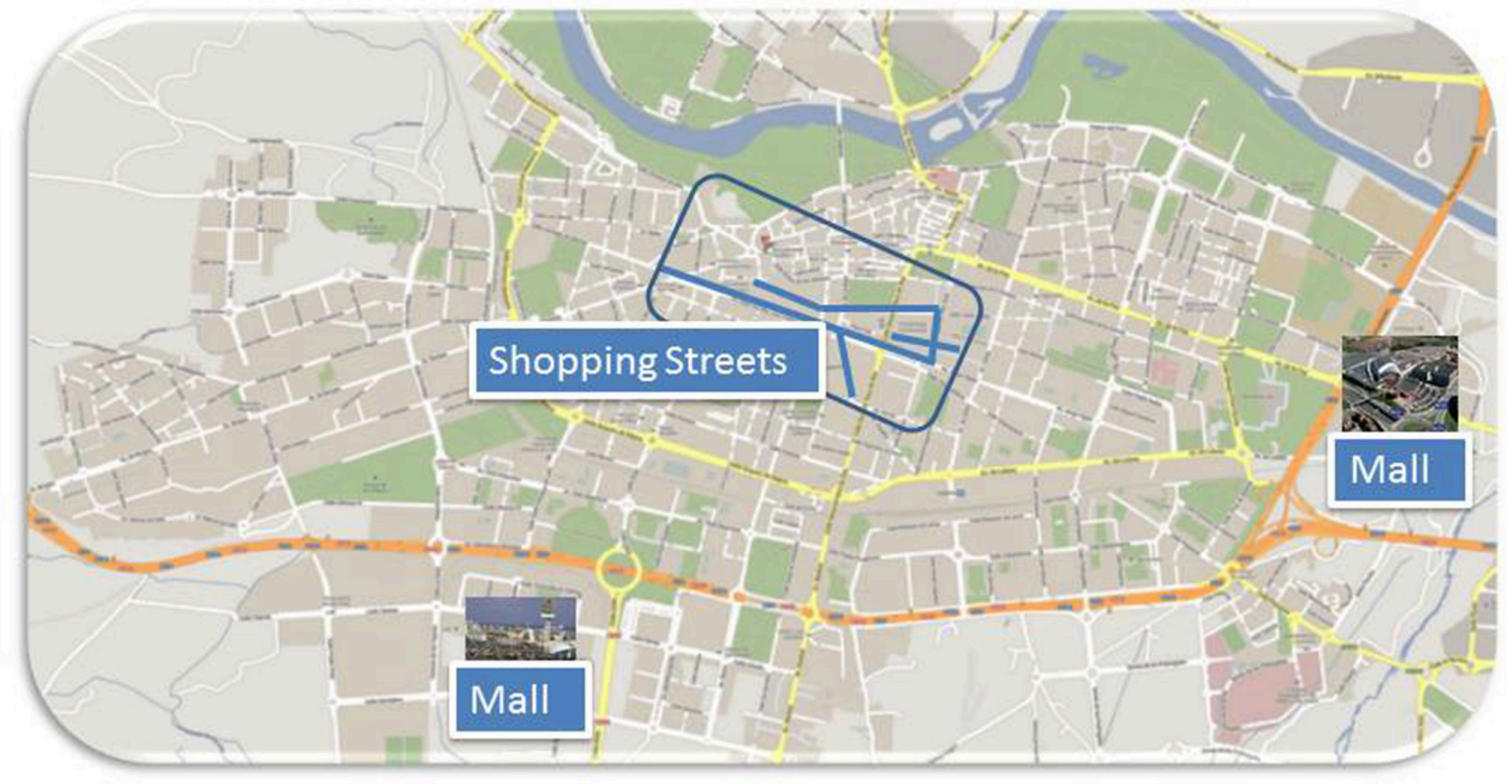
**Map of study area (Logroño—Spain)**.

Figure 2 shows our study area. The types of establishments located in the shopping streets of Logroño are mainly specialized small businesses in which the owner and their family serve the customer directly. On the outskirts of the city, two malls characterized by larger shops owned by large companies, a supermarket, and several category killers serve as the primary generators of customer traffic (Table 1).

**Table 1:** **Characteristics of the study area and sample**.

Universe	Individuals	
Sampling procedure	Quota sampling by gender and age	
Data collection	Personal in-home interviews	
Study area	Logroño, Spain	
Study area characteristics^*^:	Shopping streets	Malls
Number of shops	336	116
Size	112.36 m^2^	594.74 m^2^
Ownership:		
Sole proprietor	70.0%	5.2%
Company	30.0%	94.8%
Date of fieldwork	March–April 2013	
Sample size	220 Individuals	
Sample characteristics		
Gender	Male	43.2%
	Female	56.8%
Age	Age 25 and under	35.1%
	26 to 65 years old	41.5%
	Over 65 years old	28.3%

* Source: Data are based on a 2011 survey of retailers by Cámara de Comercio e Industria de La Rioja.

We obtained information from a quota sampling of 220 people. We considered the representativeness of the sample, establishing age and gender quotas. The method used was personal in-home interviews.

### Method

To learn about the importance of personal attention in the shopping experience, we conducted a survey using open-response questions. We asked respondents whether they preferred going shopping at a mall or a shopping street and why. The aim was to find out, indirectly and without biasing the respondents, whether personal attention is the main motivation for choosing the shopping location (H_1_). In order to contrast this hypothesis we used a binary logistic regression, applying wall method.

### Results

Of those surveyed, 54.09% reported preferring malls, 34.55% opted for shopping streets, and the rest (11.36%) reported having no preference between the two. Malls were generally preferred by young people (78.08%) and adults (44.18%) and by individuals of both sexes. However, their appeal was greater for men (55.91%) than for women (52.76%). Older people (49.18%) preferred shopping streets, and their appeal was greater for women (38.58%) than for men (29.03%).

When asked why, the respondents gave 352 reasons, which we then grouped into six primary motives. With regard to *shopping streets*, the motives stated by those surveyed, in order of importance, are as follows:

1. *Personal attention* (43.26%): this motive includes polite and courteous attention, advice, individualized attention, personal relationship, and service attitude.
2. *Spatial convenience* (29.69%): it refers to comfort and closeness to the establishment.
3. *Commercial offer* (12.16%): this motive includes fresher products and a greater variety of stores and products.
4. *Solidarity* (6.79%): this refers to being supportive of small business located in *shopping streets.*
5. *Entertainment* (4.05%): the pleasure of walking around and seeing things.
6. Negative attitude toward malls (4.05%).

With regard to *malls*, the motives stated by those surveyed, in order of importance, are as follows:

1. *Commercial offer* (49.15%): this is a motive for choosing a mall when shopping for consumer goods. Statements such as “big shopping,” “monthly shopping,” “I can find everything,” or “there are more things in a mall than in the neighborhood.” In addition, there is another key concept: *wide offer*.
2. *Spatial convenience* (28.81%): the mall is viewed as a place in which everything is in the same place or different stores are in the same place.
3. *Price* (13.56%): it is cheaper and there are bargains.
4. *Accessibility* (8.56%): it refers to opening hours, easy access, and easy parking.

Finally, we encountered buyers who did not have a clear preference, that is, those who shop both in shopping streets and at malls. These buyers reported different priorities:

1. *Commercial offer* (80.00%): the buyer who wants to see everything and the buyer who shops according to product categories.
2. *Price* (15.00%): wherever it is cheaper and there are bargains.
3. *Convenience* (5.00%): the buyer who organizes shopping on the basis of what is best for them at a given moment.

We analyze the explanatory relationship between the dichotomous dependent variable (preference between shopping streets or malls) and the independent variables (the eight most cited motives). Table 2 shows the logistic regression results. Goodness of fit were adequate: Nagelkerke's Pseudo R square = 45.6% and percentage correctly classified = 74.9%. Regarding the multicollinearity analysis the major variance inflation factor (VIF) of logit is 1.23 (corresponds to commercial offer).

**Table 2:** **Logit binomial for calculating the probability of preference between shopping streets or malls**.

	**Beta**	**Wald**	**Sig**.	**Exp. (B)**
Personal attention	3.23	9.56	0.00	25.32
Commercial offer	−2.02	22.70	0.00	0.13
Prices	−2.11	7.20	0.01	0.12
Constant	0.03	0.01	0.91	1.03
Goodness of fit	Nagelkerke's Pseudo R square = 45.6%			
	Percentage correctly classified = 74.9%			

Results show that people more likely to go to the shopping streets are those who seek personal attention. On the other hand, people more likely to go to the malls are those who look for a wide commercial offer with good price.

### Discussion

The results of this survey indicate that the main strength of retailers located in shopping streets is personal attention. Thus, we can accept H_1_. No respondents who preferred malls mentioned this motive when explaining their choice.

The strengths of malls are their wide offer and prices. Spatial convenience appears to be an important factor in both contexts, though with a different meaning. In shopping streets, it means spatial proximity. In malls, it means finding everything the customer needs in one place. In conclusion, the motivations behind choosing one retailer environment over the other are different. In addition, the study enables us to infer certain connotations about the importance of personal attention.

## Study 2. service quality incorporating personal attention in small retailers in shopping streets

The potential for personal attention to serve as a competitive advantage for small retailers located in shopping streets has led us to analyze service quality in greater depth. The objective of this second study is to learn what the most important components of personal attention are for consumers who choose small retailers.

### Literature review

#### Expectations in service quality

Expectations play a significant role in determining customer perceptions and satisfaction. Accordingly, retailers seek to manage customers' service expectations (Mitra and Fay, 2010). The literature on expectations is broad, and many ways of understanding and studying expectations have been found. After a detailed review, we have classified expectations into ten types, which we have then grouped into four main approaches: (1) comparison, (2) ideal amount, (3) levels, and (4) point of assessment (see Table 3).

**Table 3:** **Classification of expectations**.

**1. The Comparison Approach:** This refers to the use of service quality expectations to compare competitive brands. We can differentiate between:					
*(1a) Normative expectations* represent the level of service that a person believes a supplier should provide to offer excellent quality when it comes to a specific service, carrying out a realistic and feasible assessment. For example, if a person chooses a specialty luxury clothing shop to buy a dress for a special occasion, the question we ask, What level of information about the dress should the employees of a luxury clothing boutique provide?		*(1b). Equitable or deserved expectations* represent the level of service that a customer believes he or she should receive, taking into account the expenses borne—that is, what the customer considers fair. For example, the quality of service a buyer considers fair when purchasing an €800 dress in a boutique dress shop in Logroño's shopping street district. In this example, if a person spends €800, the question for an attribute might be, What would be a fair level of information about the dress to receive?		*(1c) Predictive expectations* represent the objective calculation that a person carries out regarding what he or she actually expects to receive from a supplier in a specific situation. For example, what level of service a person actually expects employees of a luxury boutique in Logroño to provide when she goes to buy a dress for a special occasion. The question in this sense would be, What level of information about the dress does this person actually hope to receive in this store?	
Source: (Miller, 1977; Oliver, 1980; Cadotte et al., 1987; Oliver and Winer, 1987; Bitner, 1990; Boulding et al., 1993; Zeithaml et al., 1993; Clow et al., 1997; Johnson and Mathews, 1997; Clow et al., 1998; Hamer et al., 1999; Kopalle and Lehmann, 2001; Kalamas et al., 2002; Anderson and Salisbury, 2003; Dean, 2004; Higgs et al., 2005; Evans et al., 2008; Mitra and Fay, 2010; Benedicktus, 2011; Lin and Wu, 2011; Yip et al., 2011; Golder et al., 2012; Yang et al., 2013; Hung, 2015; Hung et al., 2015).					
**2. The Ideal Amount Approach:** This refers to what the customer considers an ideal level of service. We can differentiate between:					
*(2a) Vector expectations* refer to attributes for which the ideal amount that the customer requires is *infinite*; therefore, the customer never reaches his or her maximum utility (e.g., an optician's level of knowledge).		*(2b) Ideal point expectations* refer to attributes for which the ideal amount that the customer requires is *finite* (e.g., the temperature of a store). A further differentiation has been made within this ideal point expectation regarding expectations for which the ideal finite amount is feasible (i.e., feasible ideal point expectations) and those that cannot be reached by any supplier (i.e., classic ideal point expectations).			
Source: (Miller, 1977; Woodruff et al., 1983; Parasuraman et al., 1985; Teas, 1993; Zeithaml et al., 1993; Parasuraman et al., 1994b; Clow et al., 1997; Higgs et al., 2005; Tsai et al., 2011; Golder et al., 2012).					
**3. The Levels Approach**: This refers to different levels of expectations that set the limits for the *interval of tolerance* allowed in the assessment of the service. We can differentiate between:					
*(3a) Desired expectations* represent the highest level of performance that a consumer considers can be reached by the suppliers of a product category (e.g., I wish I could pay by mobile phone in the store).			*(3b) Adequate expectations* reflect the minimum service quality that a consumer believes should be expected from the suppliers of a product category (e.g., I should at least be able to pay with a credit card at the store).		
Source: (Miller, 1977; Parasuraman et al., 1991, 1993, 1994a; Johnson and Mathews, 1997; Hamer et al., 1999; Bebko, 2000; Walker and Baker, 2000; Higgs et al., 2005; Nadiri and Hussain, 2005; Yap and Sweeney, 2007; Nadiri, 2011).					
**4. The Point-of-Assessment Approach:** This refers to the point at which the customer creates his or her expectations of service quality. We can differentiate between:					
*(4a) Pre-encounter expectations* are those that a person has formed before the service experience starts. For example, before going to a new luxury store, the customer has high expectations regarding its decor.			*(4b) Intra-encounter expectations* represent the quality expectations of a service that has already started. For example, when the same customer sees the facade of the new luxury store in disrepair, his or her expectations change. Upon entering the store and seeing the decor to be of high design, expectations are modified again.		
Source: (Oliver, 1980; Bitner, 1990; Bitner et al., 1990; Zeithaml et al., 1993; Mittal and Lassar, 1996; Johnson and Mathews, 1997; Clow et al., 1998; Hamer et al., 1999; Oliver and Burke, 1999; Dawar and Pillutla, 2000; Choi and Mattila, 2008).					

Consumers use *expectations* in service quality to compare competing offers (Oliver, 1977, 1980; Cadotte et al., 1987; Oliver and Burke, 1999; Andreassen, 2000). But what *type of expectations* do customers use when it comes to assessing distributors? When trying to answer this question, we find that there is no clear consensus on the topic (Zeithaml et al., 1993; Walker and Baker, 2000).

On the one hand, some research has argued that the service quality a customer receives can be measured using normative expectations and that predictive expectations are more appropriate for measuring customer satisfaction (Boulding et al., 1993; Zeithaml et al., 1993; Dean, 2004; Higgs et al., 2005). On the other hand, several researchers believe that using other types of expectations is appropriate; however, normative and/or predictive expectations are included their work as well (Golder et al., 2012).

Because our framework is related to the identification of potential competitive advantages for shopping streets, we chose the comparison approach for the analysis and classification of expectations (Table 3). This approach distinguishes and conceptualizes three types of service quality expectations to compare competitive brands: normative service quality expectations, equitable or deserved quality expectations, and predictive quality expectations.

Normative expectations represent an excellent level of service quality that a person believes a supplier should realistically and feasibly offer for a specific service—that is, what the customer thinks it should be. These expectations are usually related to a particular category of service.

Equitable expectations are defined as the equity level of service the customer feels the seller must supply, taking into account the costs incurred. Under this perspective, equitable expectations are critically determined by a personal assessment of the potential rewards vs. costs.

Predictive expectations represent the calculation a person performs to determine what he or she really expects a supplier to provide in a particular situation.

Of these three types of expectation (normative, equitable, and predictive), we decided to measure only normative and predictive expectations. We leave equitable or deserved expectations to future work, because we would need to take into account the costs the customer incurs, and our empirical study does not analyze actual purchases.

We characterize normative expectations as being more stable over time in the customer's mind (Johnson and Mathews, 1997; Clow et al., 1998). Moreover, predictive expectations are linked to the existence of the next service encounter, while normative expectations do not require any temporal proximity of the service.

#### The importance of the attributes of service quality (incorporating personal attention)

Another aspect to consider in the assessment of service quality is the *importance* of each attribute used to measure it. Teas (1993) proposes an assessment model for the quality received, differentiating between the importance of the attributes of service quality and the ideal expectations in service quality. Parasuraman et al. (1991) analyze the importance of the dimensions of service quality.

#### Relationships between service quality expectations and the importance of the attributes of service quality (incorporating personal attention)

We believe that it is important to know the expectations customers have regarding the various components of personal attention. However, customers seem to obtain simultaneous information about how important each component is. In this regard, we analyze whether normative expectations, predictive expectations, and importance are different concepts in customers' minds. We propose the following hypotheses:

H_2_: Customers are able to differentiate between normative and predictive expectations of service quality, taking into account personalization.
H_3_: Customers are able to differentiate between normative expectations and the importance of the attributes of service quality, taking into account personalization.
H_4_: Customers are able to differentiate between predictive expectations and the importance of the attributes of service quality, taking into account personalization.

### Data

#### Data collection

A face-to-face survey, applying the SERVQUAL-P scale developed by Mittal and Lassar (1996), which is an adaptation of the SERVQUAL scale developed by Parasuraman et al. (1991), was conducted on a sample of individual customers in a city in northern Spain. Respondents participated voluntarily without any compensation.

For consumers to be able to value predictive expectations, they must have a specific small store in mind. Therefore, we chose a group of stores that represent the most important sectors of the area of study (fashion and accessories, footwear, furniture and decoration, computer stores, gift stores, opticians, and travel agencies).

To measure normative expectations, predictive expectations, and the importance of the attributes of service quality, we applied a sequential process. For each attribute (observable variable), respondents rated on a scale from 0 to 10 the level of service that a store should offer for it to provide excellent service (normative expectations), the service actually expected (predictive expectations), and the importance of each item on the scale (importance). For example, for attribute 9 (“everyone at this retailer is polite and courteous”) we obtained the following: what it should be, what respondents actually expect of the service quality, and how important each item is for the customer.

#### Sample characteristics

Overall, 1088 questionnaires were collected in December 2013, but as some had to be eliminated because they were not complete, 974 usable questionnaires were obtained.

Table 4 shows that the sample of customers reproduces the structure of the population by sex and age categories.

**Table 4:** **Characteristics of Study 2**.

Universe	Individuals		
Sampling procedure	Quota sampling by gender and age		
Data collection	Personal in-home interviews		
Study area	Logroño, Spain		
Date of fieldwork	December 2013		
Sample size	974 individuals		
Sample characteristics			
		Sample%	Official average population%
Gender	Male	46.6	47.9
	Female	53.4	52.1
Age	≤25	28.3	25.7
	26–40	26.0	23.4
	41–60	28.4	29.7
	≥61	17.3	21.2

### Study 2 analysis A

We want to know how important 16 service quality attributes (incorporating personalized attention) are to the customers and the expectations they have regarding those attributes. Previously, we analyzed whether customers perceive differences between normative expectations, predictive expectations, and importance.

#### Method

To determine whether consumers perceive any differences between *normative expectations, predictive expectations*, and *importance* in the attributes of service quality, we performed 2 × 2 contrasts. Then, we obtained the underlying dimensions of the SERVQUAL-P scale for normative expectations, predictive expectations, and importance. To achieve this, we applied exploratory factor analyses, and with the dimensions obtained we applied confirmatory factor analyses to validate and confirm the factors.

#### Results

In the first stage, our results show profound differences between the average values of normative expectations and predictive expectations (Table 5). The parametric test (*t*-test) and the non-parametric test (Wilcoxon) reflect significant statistical differences, with a *p*-value lower than 0.01 for all variables.

**Table 5:** **Significant statistical differences**.

**Items**	**Average values^a^**			**NE/PE^b^**		**NE/Imp**		**PE/Imp**	
	**NE**	**PE**	**Imp**	**Sig.T**	**Sig.W**	**Sig.T**	**Sig.W**	**Sig.T**	**Sig.W**
1. Provides the service as promised	8.94	8.37	9.01	00.00	00.00	00.05	00.04	0.00	0.00
2. Is dependable in handling customers' service problems	9.12	8.38	9.06	0.00	0.00	00.05	00.12	0.00	0.00
3. Performs the service right the first time	8.93	8.18	8.78	0.00	0.00	00.00	00.00	0.00	0.00
4. All employees are well-trained and knowledgeable	9.25	8.37	9.01	0.00	0.00	00.00	00.00	0.00	0.00
5. The store employees provide prompt service	8.72	7.89	8.43	0.00	0.00	00.00	00.00	0.00	0.00
6. The store employees are always willing to help you	9.17	8.43	8.96	0.00	0.00	00.00	00.00	0.00	0.00
7. The store employees are always ready to respond to your requests	9.01	8.17	8.70	0.00	0.00	00.00	00.00	0.00	0.00
8. The store employees give customers individual attention	8.65	8.05	8.43	0.00	0.00	00.00	00.00	0.00	0.00
9. Everyone at the store is polite and courteous	9.33	8.69	9.17	0.00	0.00	00.00	00.00	0.00	0.00
10. The store employees display personal warmth in their behavior	7.54	7.00	7.16	0.00	0.00	00.00	00.00	0.00	0.00
11. All the persons working at the shop are friendly and pleasant	9.12	8.44	8.94	0.00	0.00	00.00	00.00	0.00	0.00
12. The store employees take the time to know you personally	7.36	6.65	6.97	0.00	0.00	00.00	00.00	0.00	0.00
13. The store has modern-looking equipment	9.01	8.62	8.66	0.00	0.00	00.00	00.00	0.37	0.43
14. The store's physical facilities are visually appealing	8.59	7.98	8.25	0.00	0.00	00.00	00.00	0.00	0.00
15. The store's employees have a neat and professional appearance	8.95	8.47	8.70	0.00	0.00	00.00	00.00	0.00	0.00
16. Materials associated with the service (such as pamphlets or statements) are visually appealing at the store	8.16	7.60	7.70	0.00	0.00	00.00	00.00	0.02	0.01

a NE, normative expectations; PE, predictive expectations; Imp, importance of the attribute.

b Sig.T, p-value of the t-test; Sig.W, p-value of the Wilcoxon test.

If we compare the normative and predictive expectations with the importance of the attribute, we also observe profound differences in 15 of the 16 variables (*p* ≤ 0.05).

In the second stage, the results of the exploratory factor analyses conducted on normative expectations and predictive expectations show three dimensions. For importance, we obtain four dimensions (Appendix A in Supplementary Material).

To obtain the solution of the three confirmatory factor analyses, we carried out a series of modifications. We applied the Lagrange multiplier test and calculated the Wald statistic, which evaluates the effect of freeing (or not) a group of parameters simultaneously (Hair et al., 1999). We considered the convergence of parameters in the factors to respecify the model^3^.

With regard to goodness-of-fit indexes on the three scales, the results were satisfactory (Table 6). The composite reliability coefficient shows values >0.7 (Appendix B Supplementary Material). With regard to convergent validity, the indicators converge in the factors assigned (standardized lambda parameters >0.5 and significant). The average variance extracted is ≥0.5 for all factors. Regarding discriminant validity (Table 7), the covariance between factors indicates that they differ from each other in each model. The confidence interval for the covariance value does not include the value of 1, and therefore there are no covariance issues among the factors involved.

**Table 6:** **Goodness-of-fit Indexes of each model**.

**Index^a^**	**Recommended value**	**Model 1: Normative expectations**	**Model 2: Predictive expectations**	**Model 3: Importance of the attribute**
BBNFI	>0.90	0.97	0.96	0.97
BBNNFI	>0.90	0.96	0.96	0.97
CFI	>0.95	0.98	0.97	0.98
Robust CFI	>0.95	0.95	0.96	0.95
GFI	>0.90	0.97	0.97	0.98
AGFI	>0.90	0.94	0.94	0.96
	<0.08	0.06	0.06	0.05
RMSEA		Confidence interval	Confidence interval	Confidence interval
		(0.05–0.07)	(0.05–0.07)	(0.04–0.06)

a BBNFI, Bentler–Bonett normed fit index; BBNNFI, Bentler–Bonett non-normed fit index; CFI, comparative fit index; GFI, goodness-of-fit index; AGFI, adjusted goodness-of-fit index; RMSEA, root mean square error of approximation.

**Table 7:** **Analysis of discriminant validity**.

**Factors involved**	**Covariance**	**Standard error**	**Confidence interval**		**Value outside the interval**
**MODEL 1: NORMATIVE EXPECTATIONS**					
F1–F2	0.57	0.03	0.51	0.63	1
F1–F3	0.20	0.04	0.12	0.28	1
F2–F3	0.40	0.04	0.32	0.49	1
**MODEL 2: PREDICTIVE EXPECTATIONS**					
F1–F2	0.51	0.03	0.45	0.57	1
F1–F3	0.46	0.04	0.38	0.54	1
F2–F3	0.47	0.04	0.39	0.55	1
**MODEL 3: IMPORTANCE OF THE ATTRIBUTE**					
F1–F2	0.34	0.04	0.26	0.42	1
F1–F3	0.12	0.04	0.04	0.20	1
F1–F4	0.50	0.04	0.42	0.58	1
F2–F3	0.47	0.05	0.37	0.57	1
F2–F4	0.53	0.05	0.43	0.63	1
F3–F4	0.34	0.05	0.24	0.44	1

From Table 7, we can identify three factors in the normative expectation (Model 1) and predictive expectation (Model 2) models: “service attitude and trust” (F1), “store appeal” (F2), and “personal relationship” (F3). Our results show that the dimensions of normative and predictive expectations are essentially the same. The only difference is that in F1 (service attitude and trust) of the predictive expectations, there is one additional item: “well-trained and knowledgeable employees.” With regard to the importance scale (Model 3), the dimensions obtained were “trust” (F1), “store appeal” (F2), “personal relationship” (F3), and “courteous attention” (F4).

When we performed the confirmatory factor analysis, the fundamental difference between the two expectation scales (normative and predictive) and the importance scale is that in the latter scale, there is a new dimension—“courtesy in the attention” (F4)—in which the variables “polite employees” and “friendly employees” are integrated. We removed these two variables from the predictive and normative expectation scales because the lambda factor was <0.6.

Furthermore, as the exploratory factor analysis (Appendix A in Supplementary Material) shows, they do not have a clear assignment. In addition, we checked whether the model could be improved by introducing a dimension with these two variables. The result was a model with a poorer fit.

For the importance scale, respondents are clearer that there is a dimension relating to polite and friendly behavior (the dimension's attributes covary with one another, but not with attributes of other dimensions).

#### Discussion

Our results show that there are significant differences between normative expectations (what it should be) and predictive expectations (what they actually expect the service quality to be). However, the underlying structure, which we obtained from the factorial analysis, is essentially the same. In this sense, we can partially accept H_2_. With regard to the comparison between normative and predictive expectations and importance, differences arise both in the observable variables and in the structure of the underlying dimensions. As a result, we can accept H_3_ and H_4_.

### Study 2 analysis B

In their SERVQUAL-P scale, Mittal and Lassar (1996) establish four dimensions (reliability, responsiveness, personalization, and tangibles). In the personalization dimension, they include the following aspects: “the store employees show personal warmth in their behavior,” “the employees are polite and courteous,” “the employees are friendly and pleasant,” and “they take their time to know the customer personally.” However, our results pertaining to the importance of the attributes show that the personalization dimension is divided into two subdimensions: one related to courteous attention and another to personal relationship. These results encouraged us to study these dimensions in greater detail.

#### Method

We analyzed the average value of the attributes included in the dimensions of the importance scale for service quality. Then, we applied a sequential cluster analysis to examine whether any dimension stands out in any segment.

#### Results

The average values of the attributes included in each dimension (Table 8) provide evidence that the most important factors in the average score are trust (8.95) and courteous attention (8.94). These two factors are followed by store appeal (8.22). The least important factor is personal relationship (7.07).

**Table 8:** **Average value of the attributes included in the dimensions of importance**.

**Dimensions of importance**	**F1: trust**	**F2: store appeal**	**F3: personal relationship**	**F4: courteous attention**
Average	8.95	8.22	7.07	8.94

It comes as a surprise that the courteous attention dimension is highly valued while the personal relationship dimension is the least important. Our question now is whether there is any group of consumers for whom personal relationships stand out as the most relevant dimension.

To classify customers into groups, we applied a hierarchical cluster analysis based on the dimensions of the importance scale. We used squared Euclidean distance as a proximity measurement and the Ward method as an algorithm for classifying. A dendrogram enabled us to establish the number of clusters and the centroids to subsequently apply the K-means method. As a result, we obtained four clusters (see Table 9), whose validation we carried out through two methods: variance analysis and discriminant analysis. The validation was satisfactory (see Appendix C in Supplementary Material).

**Table 9:** **Cluster according to importance**.

	**Group 1**		**Group 2**		**Group 3**		**Group 4**	
	**Mf^a^**	**Mi^b^**	**Mf**	**Mi**	**Mf**	**Mi**	**Mf**	**Mi**
F1: trust	1.45	9.59	0.29	9.08	−1.11	8.44	−3.60	7.41
F2: store appeal	1.86	9.27	0.27	8.32	−1.33	7.55	−4.35	5.76
F3: personal relationship	1.46	8.44	0.16	7.06	−1.02	6.21	−3.16	4.73
F4: courteous attention	1.88	9.73	0.35	9.14	−1.42	8.29	−4.50	6.92

a Mf, average value of the factor.

b Mi, average value of the factor's items on a scale from 0 to 10.

With regard to the description of the groups, we assigned a name to each cluster based on the importance the customers gave to the factors of service quality.

#### Group 1: highly concerned

These customers are most concerned about high service quality that incorporates personalized attention. This group values service quality in all its dimensions: trust, store appeal, personal relationship, and courteous attention. However, personal relationship is the least valued dimension. This group shows the least differences between the average values of the factors. The gap between the most valued factor (courteous attention = 9.73) and the least valued factor (personal relationship = 8.44) is 1.29 points. This cluster comprises 27.46% of the sample.

#### Group 2: concerned

This group represents more moderate customers when it comes to the importance placed on service quality (average values of the factors are close to 0 points). The average values of the items of each factor are high. The gap between the most valued factor (courteous attention = 9.14) and the least valued factor (personal relationship = 7.06) is 2.08 points. This group comprises 38.92% of the sample.

#### Group 3: value quality less

This group includes people who are less concerned than Groups 1 and 2 about the service quality of the store. The difference between the most valued factor (trust) and the least valued factor (personal relationship) is 2.23 points. This cluster comprises 28.00% of the sample.

#### Group 4: reject personal relationships

The people in this group have values on the scale that are close to 5 points (on a scale from 0 to 10), and they reject personal relationships (with values lower than 5 points). This group has the largest gap (2.68 points) between the most important dimension (trust) and the least important dimension (personal relationship). This is also the smallest group, representing only 5.62% of the total sample.

Overall, 66.38% of respondents valued courteous attention as the dimension of service quality that is most important, while for the rest it is the second most valued dimension. For 33.62%, the trust dimension—which includes proper service, capable of handling problems, and keeps promises—is the most important. Personal relationship is the least important factor for 100% of the respondents.

## General discussion

Consumer behavior represents one of the greatest interests of marketing scholars and business managers due to their need to adapt their companies' strategies to the new frontier. In this context, several authors (Badgett et al., 2007; Gentile et al., 2007; Grewal et al., 2009; Tynan and McKechnie, 2009; Verhoef et al., 2009; Rose et al., 2012) emphasize the importance of the shopping experience when choosing among different retailers. The interactions between retail employees and customers can have a significant impact on customers' perceptions of the organization (Tsiros and Parasuraman, 2006; Gremler and Gwinner, 2008; Lichtenstein et al., 2010; Otnes et al., 2012; Litz and Pollack, 2015). Despite the importance of this interaction, prior research has not answered the question of what customers value in terms of salesperson–customer interaction (Haas and Kenning, 2014). Thus, our study fills an existent gap on customer-salesperson relationship quality in retail.

In study 1, we analyze the primary motivations for customers going to shopping streets or malls since the relationship between a sales associate and a customer is dynamic and not universal and it varies across different services and industries (Kim and Jin, 2001). Our results show that the primary motivations for customers going to shopping streets or malls are different. Personalized attention is the most important factor cited by customers who prefer shopping streets. Therefore, this result is in line with numerous previous findings (Goodwin, 1996; Menon and Dubé, 2000; Stock and Hoyer, 2005; Schau et al., 2007; Gremler and Gwinner, 2008) that suggest the importance of the salesperson–customer interaction in defining the consumer's experience and ultimate satisfaction. However, personal attention is not as relevant for those who prefer malls. This is a source of competitive advantage for shopping streets, and it is the reason we studied consumer behavior in relation to service quality in these types of stores in greater detail.

Expectations also play a significant role in our research question. Retailers seek to manage customers' service expectations (Mitra and Fay, 2010). The literature on expectations is broad, and many ways of understanding and studying expectations have been found. We have classified expectations into ten types, which we have then grouped into four main approaches: (1) comparison, (2) ideal amount, (3) levels, and (4) point of assessment. Several research has argued that the service quality a customer receives can be measured using comparison approach (Boulding et al., 1993; Zeithaml et al., 1993; Dean, 2004; Higgs et al., 2005; Golder et al., 2012). This approach distinguishes and conceptualizes normative service quality expectations and predictive quality expectations. In Study 2 analysis A, we compare normative expectations (what the customer believes the service quality should be) and predictive expectations (what the customer expects the service quality will actually be) of the customers of shopping streets. The results demonstrate deep differences in the mean scores of each attribute of service quality (incorporating personalization). In keeping with numerous previous findings (Boulding et al., 1993; Johnson and Mathews, 1997; Higgs et al., 2005), expectations for normative expectations were generally higher than for the predictive expectations. However, the dimensions from the factor analysis are essentially the same: service attitude and trust (F1), store appeal (F2), and personal relationship (F3). These results are consistent with the findings of Kalamas et al. (2002), but contrary to the conclusions of Higgs et al. (2005). If the underlying dimensions of the factor analysis are the same under normative and predictive expectations, this means that the customers are coherent in their assessment of these expectations. In other words, they evaluate service quality (normative expectations) and satisfaction (predictive expectations) following a similar mindset.

According to Teas (1993), another aspect to consider in the assessment of service quality is the importance of each attribute used to measure it. Its comparison with normative and predictive expectations is crucial in the answer to our research question. Once it has been compared, differences arise both in the items and in the structure of the factors between expectations and importance of the attributes.

We were surprised to find that the personalized attention (in the importance scale) was divided into two subdimensions in our work: courteous attention and personal relationship. In Mittal and Lassar (1996), aspects related to courteous attention and aspects related to personal relationships were integrated in the personalization factor. Likewise, Gagliano and Hathcote (1994) also find a dimension related to personal attention that integrates aspects related to courtesy and the concern to help, though the notion of personal relationships is not included. Haas and Kenning (2014, p. 436) argue that “great service begins with showing courtesy to everyone, customers and coworkers alike.” Ulaga and Eggert (2006) identified service support and personal interaction as core differentiators in business relationships.

Our subsequent question was whether there was any group of consumers for whom personal relationship stands out as the most relevant dimension. In Study 2 analysis B, we classified the customers into four groups based on the dimensions of the importance scale.

Courteous attention is the most important factor for two of the groups, while for the other two it is the second most important factor. However, personal relationship is the least valued dimension in all groups.

Our results from Study 2 analysis B are consistent with those from Study 1 and according to Gremler and Gwinner (2008), the results show that the quality of service and the degree of personal attention are important factors in consumer behavior. In Study 1, the personal attention dimension was divided into five central concepts, one of them being personal relationship. Customers who preferred shopping streets value polite and courteous attention as well as close and personalized attention. However, only 4.09% of those surveyed commented that one of their motives is the personal relationship with the retailer. Therefore, there are consumers who like to maintain a personal relationship, but they are a minority.

## Managerial implications

This article advances the knowledge about this new consumer behavior and helps business managers, giving them guidelines to adapt their companies' strategies to the new frontier.

Urban shopping streets can be revitalized using personal attention in a professional manner, while stopping short of forging a personal relationship with the customer. With regard to consumer behavior, managers should take care to cultivate what kind of personal attention they offer their customers, recognizing that the personal relationship is the least valued factor in service quality.

Because the motivations for choosing malls vs. shopping streets are different, managers should consider the following recommendations. First, for managers of shops located in shopping streets, it is important to note that for 66.38% of the respondents, the most important aspect of service quality is courteous attention, and for 32.62% of the respondents, trust is the most important aspect. Thus, we recommend the following:

- Stores should select personnel who have the ability to develop these skills while not necessarily forging personal relationships (which is not valued). Because many of the retailers are family businesses, they tend to employ relatives as sellers. However, not all people are equally qualified and capable of exhibiting these sought-after characteristics.
- We recommend the development of personal attention skills by means of continuous training. Sales techniques and an appropriate use of personal communication can improve relationships with customers and their shopping experience. The “professionalization” of the seller can make the difference between companies. The use of mystery shoppers can be a tool to help identify potential improvements in sellers' personal communication skills.
- Another recommendation would be to carry out communication campaigns focused on highlighting the comfort of walking casually through shopping streets, the wide variety of stores, or the simple pleasure of shopping.

Second, for mall managers, we recommend building on the concept of “big shopping” as an excursion and consolidating their strength in the commercial offer. Commercial communication campaigns should stress the advantages of spatial convenience (everything in the same place) and accessibility (opening hours and parking). Young people show a clear preference for the mall for shopping but not for leisure activities, though in the malls in the study area there are also leisure activities. Our recommendation is to attract young people with appropriate entertainment proposals.

Because customers perceive differences between what should be (normative expectations) and what they actually expect (predictive expectations), management should carry out actions that aim to meet customers' normative expectations. This effort should focus on the attributes that are important to customers (importance scale).

## Limitations and further research

Although we believe our results add an important contribution to the literature, it is difficult to determine whether they are unique to retail in the area of study or whether they can be extrapolated beyond this environment. Therefore, we recommend that our study be replicated in other regions, such as the United States, India, or Germany, which have different cultures.

In our work we included all the retailers in the area of study as a whole. However, it would be worthwhile to conduct research that involves comparing various types of stores, as there may be differences for each category.

For Group 4, which rejects personal relationships, it would be worthwhile studying how this preference affects shopping behavior on the Internet to study e-commerce in greater detail.

Our results complement previous studies in that we have found new reasons to go shopping. We identify, among others, solidarity with traditional trade as a reason for opting to purchase in stores located in shopping streets. This result is connected to the world of emotions as a source of competitive advantage for traditional trade.

We agree with Puccinelli et al. (2009, p. 24), who argue that “the interpersonal nature of the interaction between the customer and employee […] may be key to customer satisfaction in the retail environment.” Moreover, retailers located in shopping streets must manage every encounter with the customer as a unique opportunity, in which personal attention should be the main tool for satisfying and building loyalty with the customer. Using this strategy to differentiate themselves from and compete with larger stores and malls, smaller businesses located in shopping streets might be able to effectively reestablish their retail niche and relevance to consumers.

## Author contributions

The four authors have equally participated in literature review, data analysis and writing of the paper.

### Conflict of interest statement

The authors declare that the research was conducted in the absence of any commercial or financial relationships that could be construed as a potential conflict of interest.

## Footnotes

^1^Specialty retailing combines the selling of goods and services to the consumer, and consumers expect knowledgeable, helpful staff to assist in the sales procedure (Gist, 1968).

^2^The salesperson–customer interaction is characterized by voluntary, dyadic interpersonal exchanges between the buyer and the seller and is traditionally understood to be the cornerstone of the retail/service customer experience (Meuter et al., 2000).

^3^As Anderson and Gerbing (1988) note, “you can obtain greater convergence of the model by respecifying one or more problematic indicators from different constructs or by excluding these parameters.” In our case, we considered it appropriate to eliminate parameters that contributed little to the factor to which they belonged (lambda < 0.6). However, to carry out any respecification of the model, we considered that the modification must be supported by theory.

## References

[B1] Anderson E. W. Salisbury L. C. (2003). The formation of market-level expectations and its covariates. J. Consum. Res. 30, 115–124. 10.1086/374694

[B2] Anderson J. C. Gerbing D. W. (1988). Structural equation modeling in practice: a review and recommended two-step approach. Psychol. Bull. 103, 411–423. 10.1037/0033-2909.103.3.411

[B3] Andreassen T. W. (2000). Antecedents to satisfaction with service recovery. Eur. J. Mark. 34, 156–175. 10.1108/03090560010306269

[B4] Badgett M. Boyce M. S. Kleinberger H. (2007). Turning Shoppers Into Advocates: The Customer Focused Retail Enterprise. IBM Institute for Business Value Report. Available online at: http://www-935.ibm.com/services/us/gbs/bus/pdf/g510-6554-03-shoppers-advocates.pdf (Accessed November 18, 2013).

[B5] Baker J. Levy M. Grewal D. (1992). An experimental approach to making retail store environmental decisions. J. Retail. 68, 445–460.

[B6] Baker J. Parasuraman A. Grewal D. Voss G. B. (2002). The influence of multiple store environment cues on perceived merchandise value and patronage intentions. J. Market. 66, 120–141. 10.1509/jmkg.66.2.120.18470

[B7] Bebko C. P. (2000). Service intangibility and its impact on consumer expectations of service quality. J. Serv. Market. 14, 9–26. 10.1108/08876040010309185

[B8] Benedicktus R. L. (2011). The effects of 3rd party consensus information on service expectations and online trust. J. Bus. Res. 64, 846–853. 10.1016/j.jbusres.2010.09.014

[B9] Bitner M. J. (1990). Evaluating service encounters: the effects of physical surroundings and employee responses. J. Market. 54, 69–82. 10.2307/1251871

[B10] Bitner M. J. Booms B. H. Tetreault M. S. (1990). The service encounter: diagnosing favorable and unfavorable incidents. J. Market. 54, 71–84. 10.2307/1252174

[B11] Boulding W. Kalra A. Staelin R. Zeithaml V. A. (1993). A dynamic process model of service quality: from expectations to behavioral intentions. J. Market. Res. 30, 7–27. 10.2307/3172510

[B12] Cadotte E. R. Woodruff R. B. Jenkins R. L. (1987). Expectations and norms in models of consumer satisfaction. J. Market. Res. 24, 305–314. 10.2307/3151641

[B13] Choi S. Mattila A. S. (2008). Perceives controllability and service expectations: influences on customer reactions following service failure. J. Bus. Res. 61, 24–30. 10.1016/j.jbusres.2006.05.006

[B14] Cisek S. Z. Sedikides C. Hart C. M. Godwin H. J. Benson V. Liversedge S. P. (2014). Narcissism and consumer behaviour: a review and preliminary findings. Front. Psychol. 5:232. 10.3389/fpsyg.2014.00232 24711797 PMC3968766

[B15] Clow K. E. Kurtz D. L. Ozment J. (1998). A longitudinal study of the stability of consumer expectations of services. J. Bus. Res. 42, 63–73. 10.1016/S0148-2963(97)00098-2

[B16] Clow K. E. Kurtz D. L. Ozment J. Ong B. S. (1997). The antecedents of consumer expectations of services: an empirical study across four industries. J. Serv. Market. 11, 230–248. 10.1108/08876049710171704

[B17] Dawar N. Pillutla M. M. (2000). Impact of product-harm crises on brand equity: the moderating role of consumer expectation. J. Market. Res. 37, 215–26. 10.1509/jmkr.37.2.215.18729

[B18] Dean A. M. (2004). Rethinking customer expectations of service quality: are call centers different? J. Serv. Market. 18, 60–78. 10.1108/08876040410520717

[B19] Evans K. R. Stan S. Murray L. (2008). The customer socialization paradox: the mixed effects of communicating customer role expectations. J. Serv. Market. 22, 213–223. 10.1108/08876040810871174

[B20] Gagliano K. B. Hathcote J. (1994). Customer expectations and perceptions of service quality in retail apparel specialty stores. J. Serv. Market. 8, 60–69.

[B21] Gentile C. Spiller N. Noci G. (2007). How to sustain the customer experience: an overview of experience components that co-create value with the customer. Eur. Manage. J. 25, 395–410. 10.1016/j.emj.2007.08.005

[B22] Gist R. R. (1968). Retailing: Concepts and Decisions. New York, NY: John Wiley & Sons.

[B23] Golder P. N. Mitra D. Moorman C. (2012). What is quality? An integrative framework of processes and states. J. Market. 76, 1–23. 10.1509/jm.09.0416

[B24] Goodwin C. (1996). Communality as a dimension of service relationships. J. Consum. Psychol. 5, 387–415. 10.1207/s15327663jcp0504_04

[B25] Gremler D. D. Gwinner K. P. (2008). Rapport-building behaviors used by retail employees. J. Retail. 84, 308–324. 10.1016/j.jretai.2008.07.001

[B26] Grewal D. Levy M. Kumar V. (2009). Customer experience management in retailing: an organizing framework. J. Retail. 85, 1–14. 10.1016/j.jretai.2009.01.001

[B27] Haas A. Kenning P. (2014). Utilitarian and hedonic motivators of shoppers' decision to consult with salespeople. J. Retail. 90, 428–441. 10.1016/j.jretai.2014.05.003

[B28] Hair J. F. Anderson R. E. Tatham R. L. Black W. C. (1999). Análisis Multivariante, 5th Edn. Madrid: Pretince Hall Iberica.

[B29] Hamer L. O. Liu B. S.-C. Sudharshan D. (1999). The effects of intraencounter changes in expectations on perceived service quality models. J. Serv. Res. 1, 275–289. 10.1177/109467059913008

[B30] Hernández T. Jones K. (2005). Downtowns in transition: emerging business improvement area strategies. Int. J. Retail Distrib. Manage. 33, 789–805. 10.1108/09590550510629392

[B31] Higgs B. Polonsky M. J. Hollick M. (2005). Measuring expectations: forecast vs. ideal expectations. Does it really matter? J. Retail. Consum. Serv. 12, 49–64. 10.1016/j.jretconser.2004.02.002

[B32] Hung K. (2015). Experiencing buddhism in chinese hotels: toward the construction of a religious lodging experience. J. Travel Tourism Market. 32, 1081–1098. 10.1080/10548408.2014.959632

[B33] Hung K. Wang S. Tang C. (2015). Understanding the normative expectations of customers toward Buddhism-themed hotels: a revisit of service quality. Int. J. Contemp. Hosp. Manage. 27, 1409–1441. 10.1108/IJCHM-12-2012-0264

[B34] Johnson C. Mathews B. P. (1997). The influence of experience on service expectations. Int. J. Serv. Ind. Manage. 8, 290–305. 10.1108/09564239710174381

[B35] Johnson M. D. Selnes F. (2004). Customer portfolio management: toward a dynamic theory of exchange relationships. J. Market. 68, 1–17. 10.1509/jmkg.68.2.1.27786

[B36] Kalamas M. Laroche M. Cézard A. (2002). A model of the antecedents of should and will service expectations. J. Retail. Consum. Serv. 9, 291–308. 10.1016/S0969-6989(02)00016-4

[B37] Kim S. Jin B. (2001). An evaluation of the retail service quality scale for U.S. and Korean customers of discount stores. Adv. Consum. Res. 28, 169–176.

[B38] Kopalle P. K. Lehmann D. R. (2001). Strategic management of expectations: the role of disconfirmation sensitivity and perfectionism. J. Market. Res. 38, 386–394. 10.1509/jmkr.38.3.386.18862

[B39] Léo P.-Y. Philippe J. (2002). Retail centres: location and consumer's satisfaction. Serv. Ind. J. 22, 122–146. 10.1080/714005055

[B40] Lichtenstein D. R. Netemeyer R. G. Maxham J. G. III. (2010). The relationships among manager-, employee-, and customer-company identification: implications for retail store financial performance. J. Retail. 86, 85–93. 10.1016/j.jretai.2010.01.001

[B41] Lin J.-S. C. and Wu, C.-Y. (2011). The role of expected future use in relationship-based service retention. Manag. Service Qual. 21, 535–551. 10.1108/09604521111159816

[B42] Litz R. A. Pollack J. M. (2015). Interfirm rivalry between small hardware stores and ‘big box’ retailers: market commonality and product mix similarity as antecedents to competitive response. J. Small Bus. Manage. 53, 436–449. 10.1111/jsbm.12071

[B43] Medway D. Warnaby G. Bennison D. Alexander A. (2000). Reasons for retailers' involvement in town centre management. Int. J. Retail. Distrib. Manage. 28, 368–378. 10.1108/09590550010337436

[B44] Mehrabian A. Russell J. A. (1974). An Approach to Environmental Psychology. Cambridge, MA: MIT Press.

[B45] Menon K. Dubé L. (2000). Ensuring greater satisfaction by engineering salesperson response to customer emotions. J. Retail. 76, 285–307. 10.1016/S0022-4359(00)00034-8

[B46] Meuter M. L. Ostrom A. L. Roundtree R. I. Bitner M. J. (2000). Self-service technologies: understanding customer satisfaction with technology-based service encounters. J. Market. 64, 50–64. 10.1509/jmkg.64.3.50.18024

[B47] Michelli J. A. (2007). The Starbucks Experience: 5 Principles for Turning Ordinary into Extraordinary. New York, NY: McGraw-Hill.

[B48] Miller J. A. (1977). Studying satisfaction, modifying models, eliciting expectations, posing problems, and making meaningful measurements, in Conceptualization and Measurement of Consumer Satisfaction and Dissatisfaction, ed Hunt H. K. (Bloomington, IN: School of Business, Indiana University), 72–91.

[B49] Mitra D. Fay S. (2010). Managing service expectation in online markets: a signalling theory of e-trailer pricing and empirical tests. J. Retail. 86, 184–199. 10.1016/j.jretai.2010.02.003

[B50] Mittal B. Lassar W. M. (1996). The role of personalization in service encounters. J. Retail. 72, 95–109. 10.1016/S0022-4359(96)90007-X

[B51] Nadiri H. (2011). Customers zone of tolerance for retail stores. Serv. Bus. 5, 113–37. 10.1007/s11628-011-0105-y

[B52] Nadiri H. Hussain K. (2005). Diagnosing the zone of tolerance for hotel services. Manag. Serv. Qual. 15, 259–77. 10.1108/09604520510597818

[B53] Naylor G. Kleiser S. B. Baker J. Yorkston E. (2008). Using transformational appeals to enhance the retail experience. J. Retail. 84, 49–57. 10.1016/j.jretai.2008.01.001

[B54] Novak T. P. Hoffman D. L. andYung Y.-F. (2000). Measuring the customer experience in online environments: a structural modeling approach. Market. Sci. 19, 22–24. 10.1287/mksc.19.1.22.15184

[B55] O'Callaghan E. O'Riordan D. (2003). Retailing at the periphery: an analysis of Dublin's tertiary city centre shopping streets (1972–2002). Int. J. Retail. Distrib. Manage. 31, 389–400.

[B56] Oliver R. L. (1977). Effect of expectation and disconfirmation on postexposure product evaluations: an alternative interpretation. J. Appl. Psychol. 62, 480–486. 10.1037/0021-9010.62.4.480

[B57] Oliver R. L. (1980). A cognitive model of the antecedents and consequences of satisfaction decisions. J. Market. Res. 17, 460–469. 10.2307/3150499

[B58] Oliver R. L. Burke R. R. (1999). Expectation processes in satisfaction formation: a field study. J. Serv. Res. 1, 196–214. 10.1177/109467059913002

[B59] Oliver R. L. Winer R. S. (1987). A framework for the formation and structure of consumer expectations: review and propositions. J. Econ. Psychol. 8, 469–499. 10.1016/0167-4870(87)90037-7

[B60] Otnes C. C. Ilhan B. E. Kulkarni A. (2012). The language of marketplace rituals: implications for customer experience management. J. Retail. 88, 367–383. 10.1016/j.jretai.2012.02.002

[B61] Paddison A. (2003). Town centre management (TCM): a case study of Achmore. Int. J. Retail Distrib. Manage. 31, 618–627. 10.1108/09590550310507740

[B62] Parasuraman A. Berry L. L. Zeithaml V. A. (1991). Understanding customer expectations of service. MIT Sloan Manage. Rev. 32, 39–48.

[B63] Parasuraman A. Zeithaml V. A. Berry L. L. (1985). A Conceptual model of service quality and its implications for research. J. Market. 49, 41–50. 10.2307/1251430

[B64] Parasuraman A. Zeithaml V. A. Berry L. L. (1993). More on improving service quality measurement. J. Retail. 69, 112–117. 10.1016/S0022-4359(05)80007-7

[B65] Parasuraman A. Zeithaml V. A. Berry L. L. (1994a). Alternative scales for measuring service quality: a comparative assessment based on psychometric and diagnostic criteria. J. Retail. 70, 201–230.

[B66] Parasuraman A. Zeithaml V. A. Berry L. L. (1994b). Reassessment of expectations as a comparison standard in measuring service quality: implications for further research. J. Market. 58, 111–124.

[B67] Polo Peña A. I. Frías Jamilena D. M. Rodríguez Molina M. Á. (2011). Impact of market orientation and ICT on the performance of rural smaller service enterprises. J. Small Bus. Manag. 49, 331–360. 10.1111/j.1540-627X.2011.00332.x

[B68] Puccinelli N. M. Goodstein R. C. Grewal D. Price R. Raghubir P. Stewart D. (2009). Customer experience management in retailing: understanding the buying process. J. Retail. 85, 15–30. 10.1016/j.jretai.2008.11.003

[B69] Reimers V. (2013). Convenience for the car-borne shopper: are malls and shopping strips driving customers away? Transp. Res. A Policy Pract. 49, 35–47. 10.1016/j.tra.2013.01.002

[B70] Reimers V. Clulow V. (2004). Retail concentration: a comparison of spatial convenience in shopping strips and shopping centres. J. Retail. Consum. Serv. 11, 207–221. 10.1016/S0969-6989(03)00038-9

[B71] Reynolds K. E. Beatty S. E. (1999). Customer benefits and company consequences of customer-salesperson relationships in retailing. J. Retail. 75, 11–32. 10.1016/S0022-4359(99)80002-5

[B72] Rose S. Clark M. Samouel P. Hair N. (2012). Online customer experience in e-retailing: an empirical model of antecedents and outcomes. J. Retail. 88, 308–322. 10.1016/j.jretai.2012.03.001

[B73] Sadahiro Y. (2000). A PDF-based analysis of the spatial structure of retailing. GeoJ. 52, 237–252. 10.1023/A:1014272208507

[B74] Schau H. J. Dellande S. Gilly M. C. (2007). The impact of code switching on service encounters. J. Retail. 83, 65–78. 10.1016/j.jretai.2006.10.008

[B75] Sersland D. R. (2015). Decoding the new consumer mind: how and why we shop and buy, by Kit Yarrow, Hoboken, NJ: John Wiley & Sons, 2014. ISBN: 978−1−118−64768−4. Psychol. Market. 32, 696 10.1002/mar.20810

[B76] Stock R. M. Hoyer W. D. (2005). An attitude-behavior model of salespeople's customer orientation. J. Acad. Market. Sci. 33, 536–552. 10.1177/0092070305276368

[B77] Teas R. K. (1993). Expectations, performance evaluation, and consumers' perceptions of quality. J. Market. 57, 18–34. 10.2307/1252216

[B78] Teller C. Reutterer T. (2008). The evolving concept of retail attractiveness: what makes retail agglomerations attractive when customers shop at them? J. Retail. Consum. Serv. 15, 127–143. 10.1016/j.jretconser.2007.03.003

[B79] Thakor M. V. Suri R. Saleh K. (2008). Effects of service setting and other consumers' age on the service perceptions of young consumers. J. Retail. 84, 137–149. 10.1016/j.jretai.2008.01.002

[B80] Tsai W. H. Hsu W. Chou W. C. (2011). A gap analysis model for improving airport service quality. Total Qual. Manage. 22, 1025–40. 10.1080/14783363.2011.611326

[B81] Tsiros M. Parasuraman A. (2006). The anatomy of service encounter evaluations: a conceptual framework and research propositions. Asian J. Market. 12, 4–22.

[B82] Tynan C. McKechnie S. (2009). Experience marketing: a review and reassessment. J. Market. Manage. 25, 501–517. 10.1362/026725709X461821

[B83] Ulaga W. Eggert A. (2006). Value-based differentiation in business relationships: gaining and sustaining key supplier status. J. Market. 70, 119–136. 10.1509/jmkg.2006.70.1.119

[B84] Verhoef P. C. Lemon K. N. Parasuraman A. Roggeveen A. Tsiros M. Schlesinger L. A. (2009). Customer experience creation: determinants, dynamics and management strategies. J. Retail. 85, 31–41. 10.1016/j.jretai.2008.11.001

[B85] Walker J. Baker J. (2000). An exploratory study of a multi-expectation framework for service. J. Serv. Market. 14, 411–31. 10.1108/08876040010340946

[B86] Woodruff R. B. Cadotte E. R. Jenkins R. L. (1983). Modeling consumer satisfaction processes using experience-based norms. J. Market. Res. 20, 296–304. 10.2307/3151833

[B87] Yang B. Kim Y. Yoo C. (2013). The integrated mobile advertising model: the effects of technology- and emotion-based evaluations. J. Bus. Res. 66, 1345–1352. 10.1016/j.jbusres.2012.02.035

[B88] Yap K. B. Sweeney J. C. (2007). Zone-of-tolerance moderates the service quality-outcome relationship. J. Serv. Market. 21, 137–148. 10.1108/08876040710737895

[B89] Yip J. Chan H. H. T. Kwan B. Law D. (2011). Influence of appearance orientation, BI and purchase intention on customer expectations of service quality in Hong Kong intimate apparel retailing. Total Qual. Manage. Bus. Excell. 22, 1105–1118. 10.1080/14783363.2011.593904

[B90] Zeithaml V. A. Berry L. L. Parasuraman A. (1993). The nature and determinants of customer expectations of service. J. Acad. Market. Sci. 21, 1–12. 10.1177/0092070393211001

