# Supplementary material for: Consumer Behavior in Shopping Streets: The Importance of the Salesperson's Professional Personal Attention
Source: Front Psychol. 2016 Feb 10;7:125. doi: 10.3389/fpsyg.2016.00125 (PMC4748058; doi:10.3389/fpsyg.2016.00125)
Supplement: Supplementary file 1 [file DataSheet1.docx]

Appendix A: Exploratory Factor Analysis of the Observable Variables

*A: The Normative Expectations Scale*

| **Variable** | | **Factor Loading** | | | | |
| --- | --- | --- | --- | --- | --- | --- |
|  |  | **F1** | **F2** | | | **F3** |
| P2.2.*should*. Capable of handling problems | | **0.748** | 0.124 | | | 0.009 |
| P2.3.*should*. Proper service | | **0.694** | 0.166 | | | 0.057 |
| P2.1.*should*. Keeps promises | | **0.668** | 0.189 | | | –0.046 |
| P2.7.*should*. Prepared to respond to requests | | **0.662** | 0.170 | | | 0.212 |
| P2.6.*should*. Prepared to help | | **0.659** | 0.199 | | | 0.156 |
| P2.4.*should*. Well-trained and knowledgeable employees | | **0.622** | 0.210 | | | 0.002 |
| P2.5.*should*. Prompt service | | **0.558** | 0.282 | | | 0.201 |
| P2.9.*should*. Polite employees | | **0.509** | **0.405** | | | –0.006 |
| P2.14.*should*. Appealing store | | 0.161 | **0.772** | | | 0.137 |
| P2.15.*should*. Employees have a neat appearance | | 0.321 | **0.692** | | | 0.117 |
| P2.16.*should*. Materials associated with the service | | 0.084 | **0.641** | | | **0.401** |
| P2.13.*should*. Retail has modern-looking equipment | | 0.258 | **0.621** | | | 0.007 |
| P2.11.*should*. Friendly employees | | **0.410** | **0.505** | | | 0.150 |
| P2.10.*should*. Show personal warmth in their behavior | | 0.032 | 0.093 | | | **0.821** |
| P2.12.*should*. Take time to know the customer personally | | 0.033 | 0.166 | | | **0.821** |
| P2.8.*should*. Employees give customers individual attention | | **0.441** | 0.150 | | | **0.480** |
| **Total Variance Explained** | | | | | | |
| **Factor** | **% of the Variance** | | | **% Accumulated** | | |
| F1. Service attitude and trust | 24.267 | | | 24.267 | | |
| F2. Store appeal | 16.374 | | | 40.641 | | |
| F3. Personal relationship | 11.938 | | | 52.578 | | |
|  | | | | | | |
| **Goodness of Fit** | | | | | | |
| Determinants | | | | | 0.006 | |
| Kaiser–Meyer–Olkin measure of adequacy | | | | | 0.905 | |
| Bartlett’s test of sphericity (approx. chi-squared) | | | | | 4,730.0 (*p* < 0.001) | |

*B: The Predictive Expectations Scale*

| **Variable** | | **Factor loading** | | | | |
| --- | --- | --- | --- | --- | --- | --- |
|  |  | **F1** | **F2** | | | **F3** |
| P2.2.*expect*. Capable of handling problems | | **0.752** | 0.127 | | | 0.019 |
| P2.3.*expect*. Proper service | | **0.740** | 0.113 | | | 0.069 |
| P2.1.*expect*. Keeps promises | | **0.726** | 0.143 | | | –0.034 |
| P2.4.*expect*. Well-trained and knowledgeable employees | | **0.685** | 0.149 | | | 0.155 |
| P2.6.*expect*. Prepared to help | | **0.678** | 0.192 | | | 0.257 |
| P2.7.*expect*. Prepared to respond to requests | | **0.661** | 0.177 | | | 0.286 |
| P2.5.*expect*. Prompt service | | **0.609** | 0.168 | | | 0.242 |
| P2.9.*expect*. Polite employees | | **0.572** | 0.365 | | | 0.108 |
| P2.11.*expect*. Friendly employees | | **0.533** | **0.416** | | | 0.237 |
| P2.8.*expect*. Employees give customers individual attention | | **0.514** | 0.171 | | | **0.410** |
| P2.15.*expect*. Employees have a neat appearance | | 0.243 | **0.741** | | | 0.083 |
| P2.13.*expect*. Retail has modern-looking equipment | | 0.188 | **0.657** | | | –0.109 |
| P2.14.*expect*. Appealing store | | 0.195 | **0.629** | | | 0.342 |
| P2.16.*expect*. Materials associated with the service | | 0.074 | **0.609** | | | 0.381 |
| P2.10.*expect*. Show personal warmth in their behavior | | 0.173 | 0.080 | | | **0.825** |
| P2.12.*expect*. Take time to know the customer personally | | 0.155 | 0.154 | | | **0.811** |
| **Total Variance Explained** | | | | | | |
| **Factor** | **% of the Variance** | | | **% Accumulated** | | |
| F1. Service attitude and trust | 27.788 | | | 27.788 | | |
| F2. Store appeal | 14.258 | | | 42.046 | | |
| F3. Personal relationship | 13.071 | | | 55.117 | | |
|  | | | | | | |
| **Goodness of Fit** | | | | | | |
| Determinants | | | | | 0.003 | |
| Kaiser–Meyer–Olkin measure of adequacy | | | | | 0.922 | |
| Bartlett’s test of sphericity (approx. chi-squared) | | | | | 5,311.91 (*p* < 0.001) | |

*C: The Importance Scale*

| **Variable** | | **Factor Loading** | | | |
| --- | --- | --- | --- | --- | --- |
|  |  | **F1** | **F2** | **F3** | **F4** |
| P2.3.*important*. Proper service | | **0.743** | 0.156 | 0.117 | –0.013 |
| P2.2.important. Capable of handling problems | | **0.702** | 0.090 | –0.082 | 0.229 |
| P2.1.*important*. Keeps promises | | **0.676** | 0.101 | –0.050 | 0.241 |
| P2.4.*important*. Well-trained and knowledgeable employees | | **0.569** | 0.115 | 0.100 | 0.297 |
| P2.5.*important*. Prompt service | | **0.527** | 0.303 | 0.262 | 0.014 |
| P2.14.*important*. Appealing store | | 0.135 | **0.789** | 0.088 | 0.081 |
| P2.16.*important*. Materials associated with the service | | 0.079 | **0.719** | 0.326 | 0.053 |
| P2.15.*important*. Employees have a neat appearance | | 0.110 | **0.651** | 0.086 | 0.371 |
| P2.13.*important*. Retail has modern-looking equipment | | 0.259 | **0.549** | –0.004 | 0.139 |
| P2.9.*important*. Polite employees | | 0.161 | 0.157 | 0.055 | **0.786** |
| P2.11.*important*. Friendly employees | | 0.115 | 0.224 | 0.140 | **0.744** |
| P2.6.*important*. Prepared to help | | **0.411** | 0.017 | 0.194 | **0.535** |
| P2.7.*important*. Prepared to respond to requests | | **0.416** | 0.175 | 0.215 | **0.465** |
| P2.10.*important*. Show personal warmth in their behavior | | 0.016 | 0.080 | **0.822** | 0.099 |
| P2.12.*important*. Take time to know the customer personally | | –0.019 | 0.198 | **0.808** | 0.071 |
| P2.8.*important*. Employees give customers individual attention | | 0.263 | 0.077 | **0.531** | 0.241 |
| **Total Variance Explained** | | | | | |
| **Factor** | **% of the Variance** | | **% Accumulated** | | |
| F1. Trust | 16.605 | | 16.605 | | |
| F2. Store appeal | 13.565 | | 30.170 | | |
| F3. Personal relationship | 12.137 | | 42.307 | | |
| F4. Courteous attention | 13.204 | | 55.511 | | |
|  | | | | | |
| **Goodness of Fit** | | | | | |
| Determinants | | | 0.014 | | |
| Kaiser–Meyer–Olkin measure of adequacy | | | 0.874 | | |
| Bartlett’s test of sphericity (approx. chi-squared) | | | 3,916.74 (*p* < 0.001) | | |

Appendix B: Analysis of the Convergent Validity and Reliability of the Three Models

|  | **Standardized Parameters >0.5 and *t*-value > 1.96** | **Composite Reliability Coefficient > 0.7** | **Average Variance Extracted ≥ 0.5** |
| --- | --- | --- | --- |
| **Model 1: Normative Expectations** | | | |
| **F1. Service Attitude and Trust** |  | 0.85 | 0.50 |
| Capable of handling problems | 0.75 (23.6) |  |  |
| Proper service | 0.61 (19.8) |  |  |
| Keeps promises | 0.73 (21.8) |  |  |
| Prepared to respond to requests | 0.74 (2.8) |  |  |
| Prepared to help | 0.63 (19.9) |  |  |
| Prompt service | 0.76 (18.7) |  |  |
| **F2. Store Appeal** |  | 0.77 | 0.52 |
| Appealing store | 0.70 (2.6) |  |  |
| Employees have a neat appearance | 0.75 (2.6) |  |  |
| Materials associated with the service | 0.71 (19.0) |  |  |
| **F3. Personal Relationships** |  | 0.72 | 0.58 |
| Show personal warmth in their behavior | 0.89 (34.3) |  |  |
| Take time to know the customer personally | 0.60 (18.3) |  |  |
| **Model 2: Predictive Expectations** | | | |
| **F1. Service Attitude and Trust** |  | 0.87 | 0.50 |
| Capable of handling problems | 0.71 (23.4) |  |  |
| Proper service | 0.70 (22.1) |  |  |
| Keeps promises | 0.73 (22.4) |  |  |
| Prepared to respond to requests | 0.76 (19.8) |  |  |
| Prepared to help | 0.72 (22.3) |  |  |
| Prompt service | 0.62 (19.0) |  |  |
| Well-trained and knowledgeable employees | 0.68 (21.6) |  |  |
| **F2. Store Appeal** |  | 0.76 | 0.52 |
| Appealing store | 0.83 (29.9) |  |  |
| Employees have a neat appearance | 0.61 (18.4) |  |  |
| Materials associated with the service | 0.70 (16.6) |  |  |
| **F3. Personal Relationships** |  | 0.73 | 0.57 |
| Show personal warmth in their behavior | 0.74 (19.1) |  |  |
| Take time to know the customer personally | 0.77 (19.8) |  |  |
| **Model 3: Importance of the Attribute** | | | |
| **F1. Trust** |  | 0.78 | 0.55 |
| Proper service | 0.60 (13.9) |  |  |
| Capable of handling problems | 0.71 (15.6) |  |  |
| Keeps promises | 0.89 (34.3) |  |  |
| **F2. Store Appeal** |  | 0.75 | 0.50 |
| Appealing store | 0.62 (17.2) |  |  |
| Materials associated to the service | 0.74 (18.3) |  |  |
| Employees have a neat appearance | 0.75 (18.3) |  |  |
| **F3. Personal Relationships** |  | 0.71 | 0.55 |
| Show personal warmth in their behavior | 0.69 (16.6) |  |  |
| Take time to know the customer personally | 0.79 (18.0) |  |  |
| **F4. Courteous Attention** |  | 0.79 | 0.55 |
| Polite employees | 0.69 (18.9) |  |  |
| Friendly employees | 0.75 (2.3) |  |  |
| Prepared to respond to requests | 0.79 (14.6) |  |  |

Appendix C. Validation of the Cluster

Table B1 shows the average values of the points obtained for the observable variables of both groups of users and the ANOVA results. The ANOVA results reflect the existence of a difference of averages between groups.

Table B1: Average Values on a 0–5 Scale Between User Groups and ANOVA Results

|  | **GROUP 1** | **GROUP 2** | **GROUP 3** | **GROUP 4** | **ANOVA Significance** |
| --- | --- | --- | --- | --- | --- |
| F1. Trust | 0.5299 | 0.8004 | –0.6627 | –2.7771 | 0.00 |
| F2. Store appeal | 1.8165 | 0.6978 | –0.7149 | –2.9595 | 0.00 |
| F3. Personal relationships | 1.6519 | –0.0644 | –0.1820 | –2.6028 | 0.00 |
| F4. Courteous attention | 1.2218 | –0.0363 | –1.0218 | –3.7251 | 0.00 |

Likewise, the multiple discriminant analysis shows the existence of a difference of averages between customers in the test of equality of averages (F) (see Table B2). In addition, we observe low values of Wilks’s lambda, and the chi-squared associated with Wilks’s lambda enables us to accept the hypothesis of differences in the points awarded to the independent variables between the groups of consumers (see Table B3). In the same way, Box’s M test yields an F-statistic of 19.19, with a significance level lower than 0.001. This enables us to reject the null hypothesis that the variance–covariance matrices do not present significant statistical differences between groups. Finally, the confusion matrix shows that 98.6% of the original grouped cases are correctly classified. All this confirms that both of the clusters obtained are different and that they are correctly identified.

Table B2: Equality Tests of the Groups’ Averages

| **Variables** | **Wilks’s Lambda** | **F-statistic** |
| --- | --- | --- |
| F1. Trust | 0.255 | 897.396* |
| F2. Store appeal | 0.138 | 1920.167* |
| F3. Personal relationships | 0.260 | 874.274* |
| F4. Courteous attention | 0.134 | 1978.774* |

*Significant at *p* < 0.01

Table B3: Wilks’s Lambda

| **Contrast of the Function** | **Wilks’s Lambda** | **Chi-Squared** | **Degrees of Freedom** | **Significance** |
| --- | --- | --- | --- | --- |
| 1 to 3 | 0.115 | 1990.913 | 12 | 0.000 |
| 2 to 3 | 0.978 | 20.477 | 6 | 0.002 |
| 3 | 0.994 | 5.512 | 2 | 0.064 |
